# A Complex Arrhythmic Phenotype in a Pediatric Patient with Variants in *SCN5A* and *KCNH2*

**DOI:** 10.3390/genes17080885

**Published:** 2026-07-29

**Authors:** David Bienjonetti-Boudreau, Camille Tremblay-Laganiere, Efthymia Gkogkou, Annick Martinez, Cecilia Gonzalez Corcia

**Affiliations:** Department of Pediatrics, Centre Hospitalier Universitaire Sainte-Justine (CHUSJ), 3175 Chemin de la Côte-Sainte-Catherine, Montreal, QC H3T 1C5, Canada; camille.tremblay-laganiere.med@ssss.gouv.qc.ca (C.T.-L.); efthymia.gkogkou@umontreal.ca (E.G.); annick.martinez.hsj@ssss.gouv.qc.ca (A.M.); cecilia.gonzalez-corcia.med@ssss.gouv.qc.ca (C.G.C.)

**Keywords:** genetics, channelopathy, *KCNH2*, *SCN5A*, digenic mutations, cardiology, arrhythmia

## Abstract

Long QT Syndrome type 2 (LQT2) and Brugada syndrome are inherited cardiac channelopathies that predispose affected individuals to ventricular arrhythmias and sudden cardiac death. We report the case of a 15-year-old male carrying two pathogenic variants: one in SCN5A and one in KCNH2, genes classically associated with Brugada syndrome and LQT2, respectively. The patient presented with an atypical phenotype characterized predominantly by atrial arrhythmias, atrioventricular (AV) conduction abnormalities, and wide complex tachycardia and sinus node disease without the characteristic electrocardiographic features of either syndrome. The phenotype was most likely driven predominantly by the SCN5A variant. Given his unpredictable episodes of high-grade conduction block and potential risk of ventricular arrhythmias, a transvenous implantable cardioverter-defibrillator (ICD) was implanted. Family screening revealed segregation of *SCN5A* and *KCNH2* variants in different relatives, each with mild or absent phenotype. This case highlights the complexity of genotype–phenotype correlations in inherited arrhythmia syndromes and the importance of individualized genetics-informed management.

## 1. Introduction

The *SCN5A* gene encodes the α-subunit of the voltage-dependent cardiac sodium channel (Nav 1.5). This channel is responsible for the inward sodium current during the initial phase of the action potential. Pathogenic variations in *SCN5A* impair Nav 1.5 function and disrupt myocardial depolarization, leading to a broad spectrum of clinical phenotypes [1]. This includes Brugada syndrome, progressive cardiac conduction disease (Lev–Lenègre syndrome), Long QT syndrome type 3 (LQT3), sinus node dysfunction, atrial fibrillation, and dilated cardiomyopathy [2].

The *KCNH2* gene encodes the pore-forming subunit of the KV11.1 potassium channel, which mediates the rapid component of the delayed rectifier potassium current (IKr). Loss-of-function variants in *KCNH2* reduce outward potassium current, leading to delayed repolarization and prolonged action potential duration—characteristic of Long QT syndrome type 2 (LQT2) [3].

We report the case of a 15-year-old carrying digenic heterozygous variants in both *SCN5A* (NM_003335.5: c.656G>A; p.Arg219His) and KCNH2 (NM_000238.4: c.2717C>T; p.Ser906Leu) genes. This case is notable because the patient’s presentation did not conform to the classical electrocardiographic or clinical phenotypes associated with either variant and raises the question of whether the KCNH2 variant modifies disease expression or represents an incidental finding, highlighting the complexity of genotype–phenotype correlations.

## 2. Case Report

We report the case of a 15-year-old Caucasian (French Canadian) male, with a weight of 86.7 kg and height of 192.4 cm, referred to our cardiology clinic in August 2023 for evaluation of palpitations and suspected supraventricular tachycardia (SVT) identified on an ambulatory Holter monitor. At that time, he had no other comorbidities and was not taking any medication. He was on a competitive high school basketball team.

**Figure 1:**
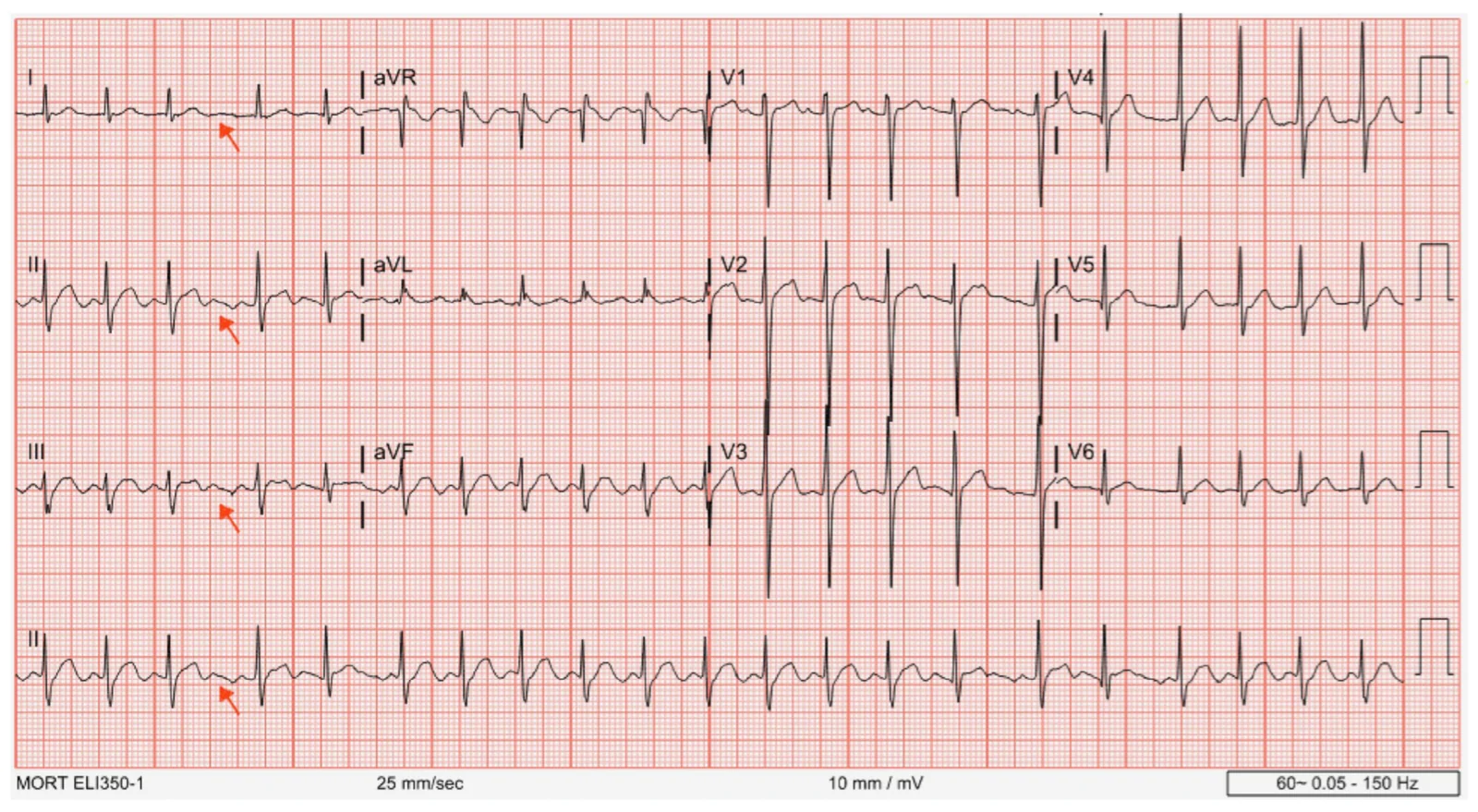
12-lead ECG at initial presentation showing a rapid atrial flutter with a ventricular rate of approximately 150 bpm and irregular RR intervals due to variable AV conduction. During periods of 1:1 AV conduction, flutter waves are difficult to identify, whereas when AV conduction decreases (after the third beat), the flutter waves become unmasked (red arrow) (August 2023).

**Figure 2:**
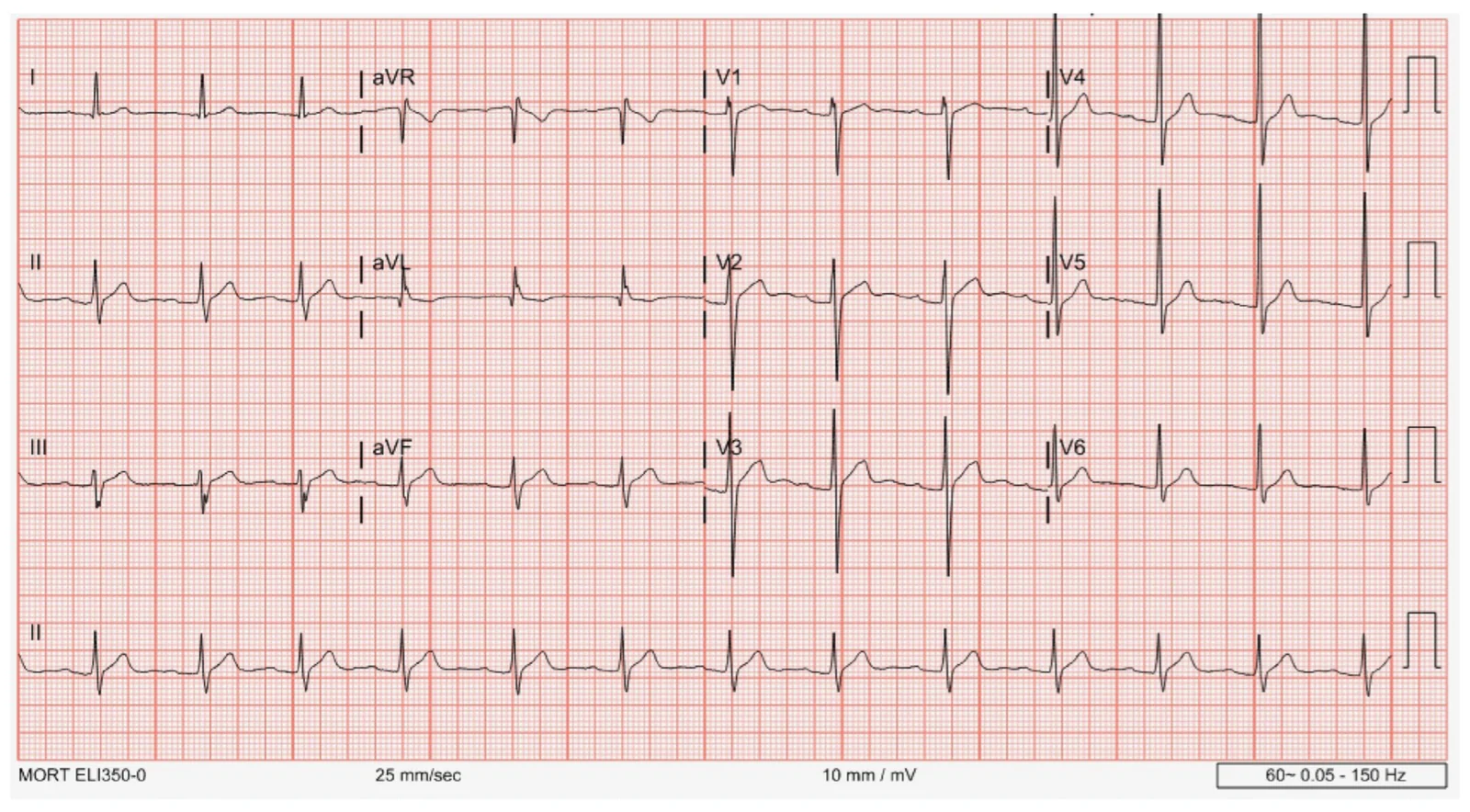
A 12-lead ECG at initial after cardioversion showing sinus rhythm of 75 bpm with first degree AV block with a PR interval of 226 msec. Interestingly the P waves are subtle, which can be suggestive of atrial myopathy (August 2023).

At initial presentation in August 2023, the patient presented with atrial flutter with a rapid and variable ventricular response, alternating between 2:1 and 3:1 AV conduction (Figure 1). He was admitted for transesophageal echocardiogram (TEE) and planned electrical cardioversion; however, spontaneous conversion to normal sinus rhythm occurred prior to the procedure. Baseline 12-lead electrocardiogram (ECG) in sinus rhythm showed first-degree AV block with a PR interval of 226 ms (Figure 2). Transthoracic echocardiography revealed normal cardiac structure and preserved biventricular function. Subsequent 24-h Holter monitoring revealed multiple symptomatic paroxysmal episodes of atrial tachycardia, prompting referral for an elective electrophysiology study (EPS).

**Figure 3:**
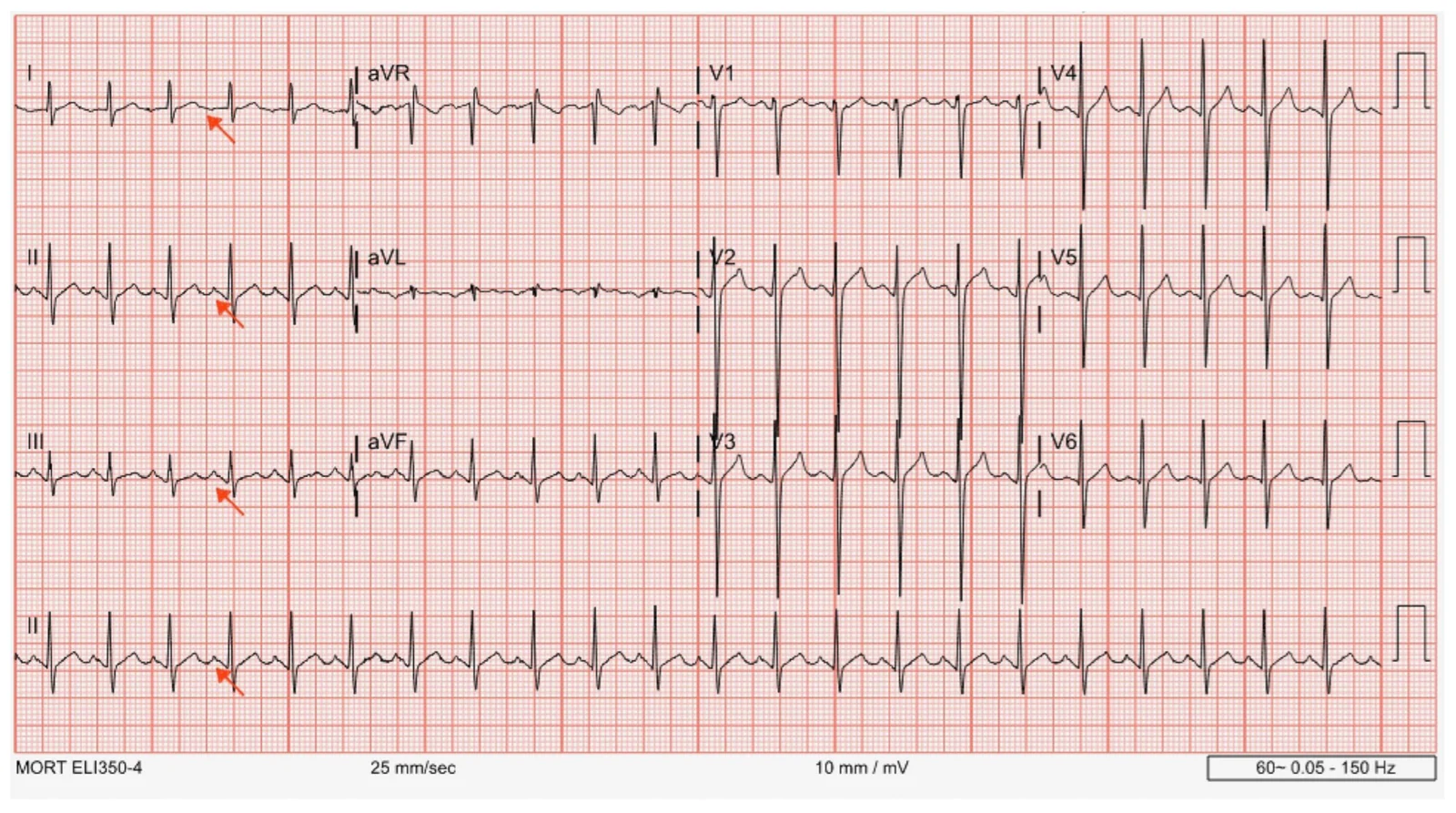
A 12-lead ECG with narrow complex QRS tachycardia, with longer RP than PR. Probable atrial tachycardia at a rate of 123 bpm. The P wave compared to the baseline EKG has a different axis (red arrows) (September 2023).

In September 2023, the patient was rehospitalized for a persistent episode of atrial tachycardia with ventricular rate of approximately 120 bpm (Figure 3). Repeated transthoracic echocardiography demonstrated mild reduced left ventricular systolic function, with an estimated left ventricular ejection fraction of 53%, and without chamber dilation. Cardiac magnetic resonance imaging (CMR) also revealed mild left ventricular systolic dysfunction, with a left ventricular ejection fraction of 48%, no late gadolinium enhancement and no evidence of arrhythmogenic right ventricular cardiomyopathy. A procainamide provocation test was negative for a Brugada ECG pattern.

During the EPS, an ectopic atrial focus originating from the left atrial appendage was identified and successfully ablated. An implantable loop recorder (ILR) was implanted for long-term rhythm monitoring.

Later in September 2023, the patient experienced recurrent atrial tachycardia, prompting rehospitalization. Propranolol therapy was initiated for arrhythmia control, and a repeated EPS was scheduled.

**Figure 4:**
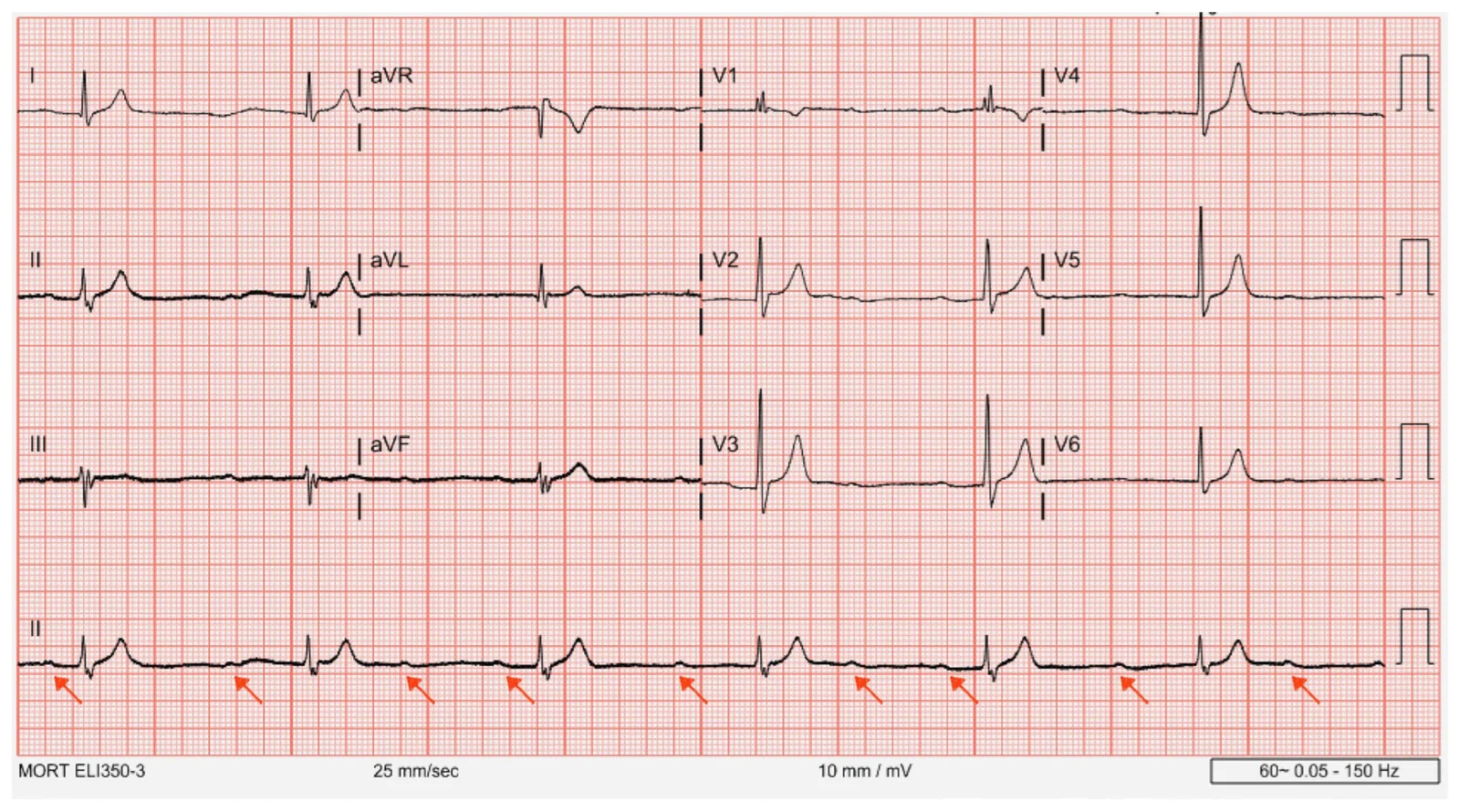
A 12-lead ECG with CHB with a slow junctional escape rhythm at 30 bpm and atrial rate around 60 bpm (red arrows). P waves are low amplitude and share the same morphology as sinus P waves. (November 2023).

During the second EPS in November 2023, four distinct atrial ectopic foci were identified. Given the multifocal nature of the arrhythmia, no ablation was performed, and medical management was continued. During the EPS, the atrial-His (AH) interval remained stable at approximately 85 ms and the His-ventricular (HV) interval at 50 ms, both within normal limits and with no mechanical AV block. Unexpectedly, the following afternoon, approximately 24 h after the procedure, the patient developed complete heart block (CHB), with a slow junctional escape rhythm at 30 bpm. (Figure 4). Although hemodynamically stable, the patient was symptomatic. Acute management included intravenous atropine followed by an isoproterenol infusion. He subsequently underwent urgent temporary transvenous pacing in VVI mode at a lower rate of 50 bpm.

**Figure 5:**
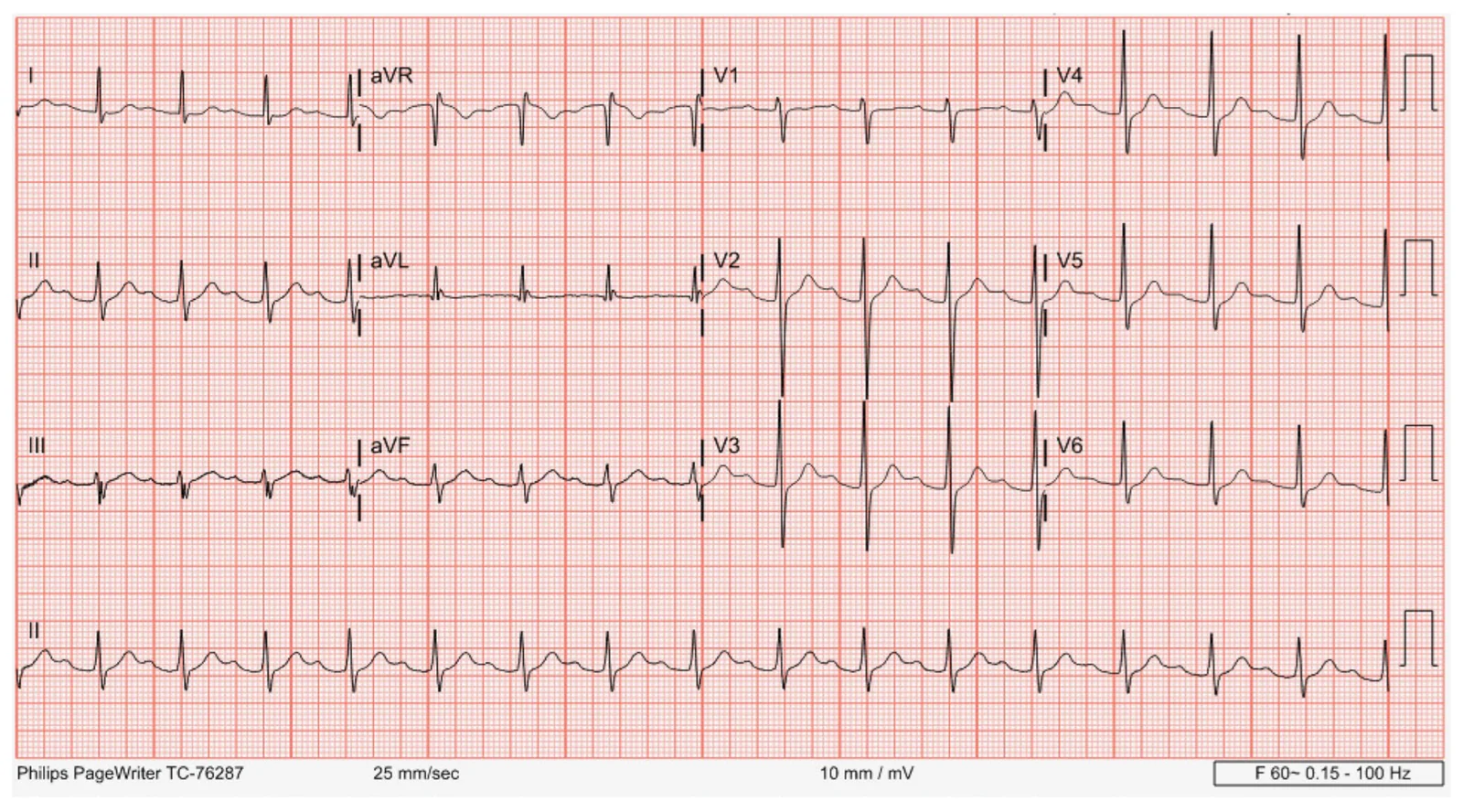
A 12-lead ECG after resolution of CHB with sinus rhythm at 104 bpm and first-degree AV block with a PR of 260–280 ms. (November 2023).

Following this episode, propranolol was immediately discontinued and withheld for several months. An intermittent high-grade AV block persisted over the following days before gradual recovery of normal AV conduction. Approximately one week after the initial episode of CHB, following recovery of normal AV conduction, an exercise stress test demonstrated a normal chronotropic response, with no recurrence of AV block or abnormal QT behavior at peak exercise and during recovery. The patient had been off propranolol for one week at the time of the exercise test. A Holter monitor performed also one week after the onset of the CHB showed occasional episodes of atrial tachycardia but no high-grade AV block. A first-degree AV block persisted with a PR interval ranging from 200 to 280 ms on serial ECGs (Figure 5).

During hospitalization, comprehensive genetic testing was performed using a sudden death gene panel at the Molecular Diagnostic Laboratory for Inherited Cardiovascular Disease of the Montreal Heart Institute using a validated next-generation sequencing (NGS) assay for unexplained cardiac arrest. Genomic DNA extracted from peripheral blood underwent target enrichment using custom Twist Bioscience probes and the Illumina DNA Prep with Enrichment protocol, followed by sequencing on a NextSeq2000 platform. The panel interrogated 74 genes (list provided in Supplementary Documents) associated with inherited arrhythmia syndromes, cardiomyopathies, and aortopathies, including analysis of coding exons, exon–intron boundaries (±10 bp), selected intronic regions, and copy-number variants with a median sequencing depth of 1004x, with reported analytical sensitivity and specificity exceeding 99% for single nucleotide variants and small insertions/deletions. Analysis included detection of single-nucleotide variants (SNVs), small insertions/deletions (InDels), and copy-number variants (CNVs) using the GRCh38/hg38 reference genome assembly. Variants were interpreted according to ACMG/AMP classification. Two heterozygous variants classified as likely pathogenic were identified: NM_003335.5 (*SCN5A*): c.656G>A; p.Arg219His, and NM_000238.4 (KCNH2): c.2717C>T; p.Ser906Leu. A detailed variant interpretation is provided in the discussion.

**Figure 6:**
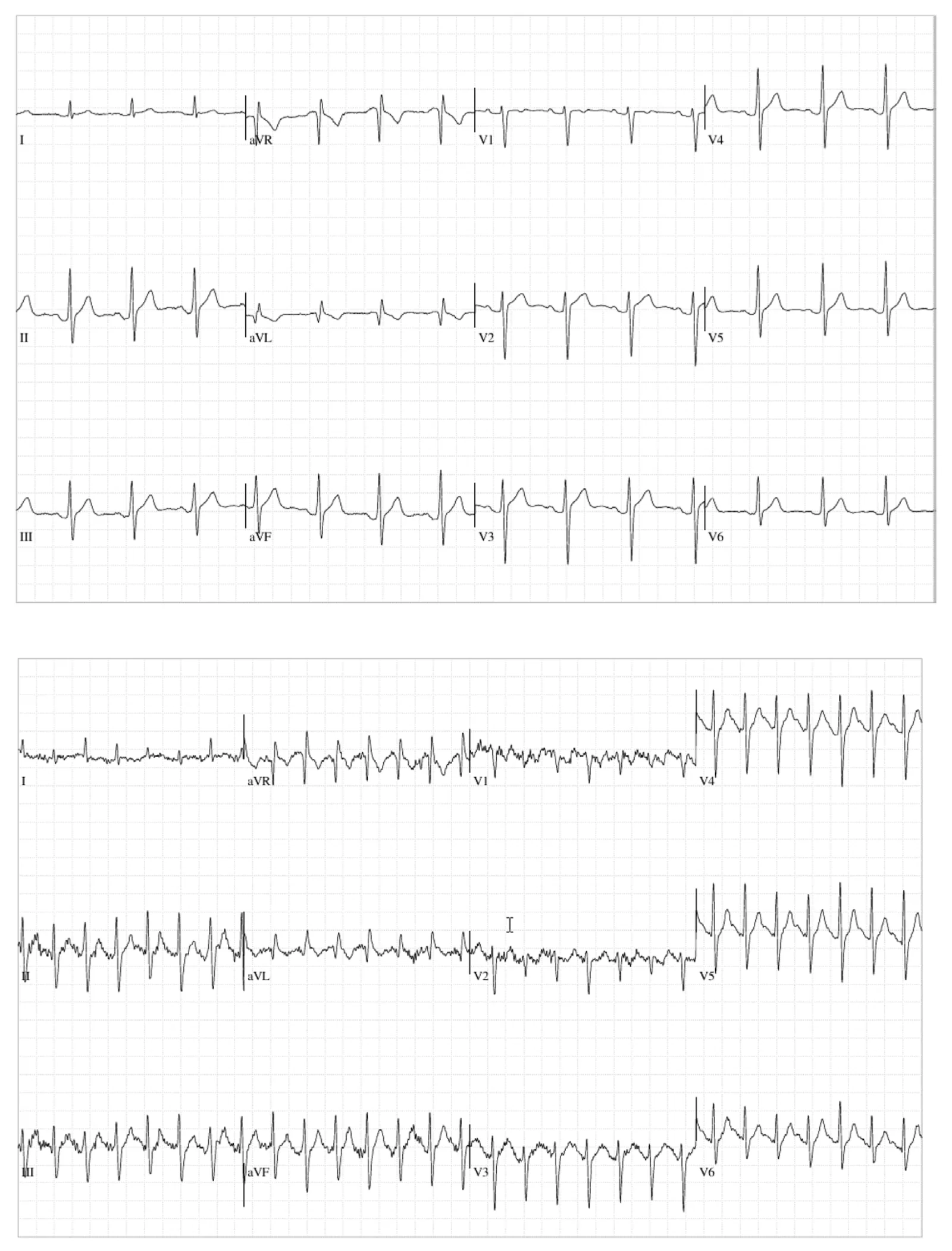

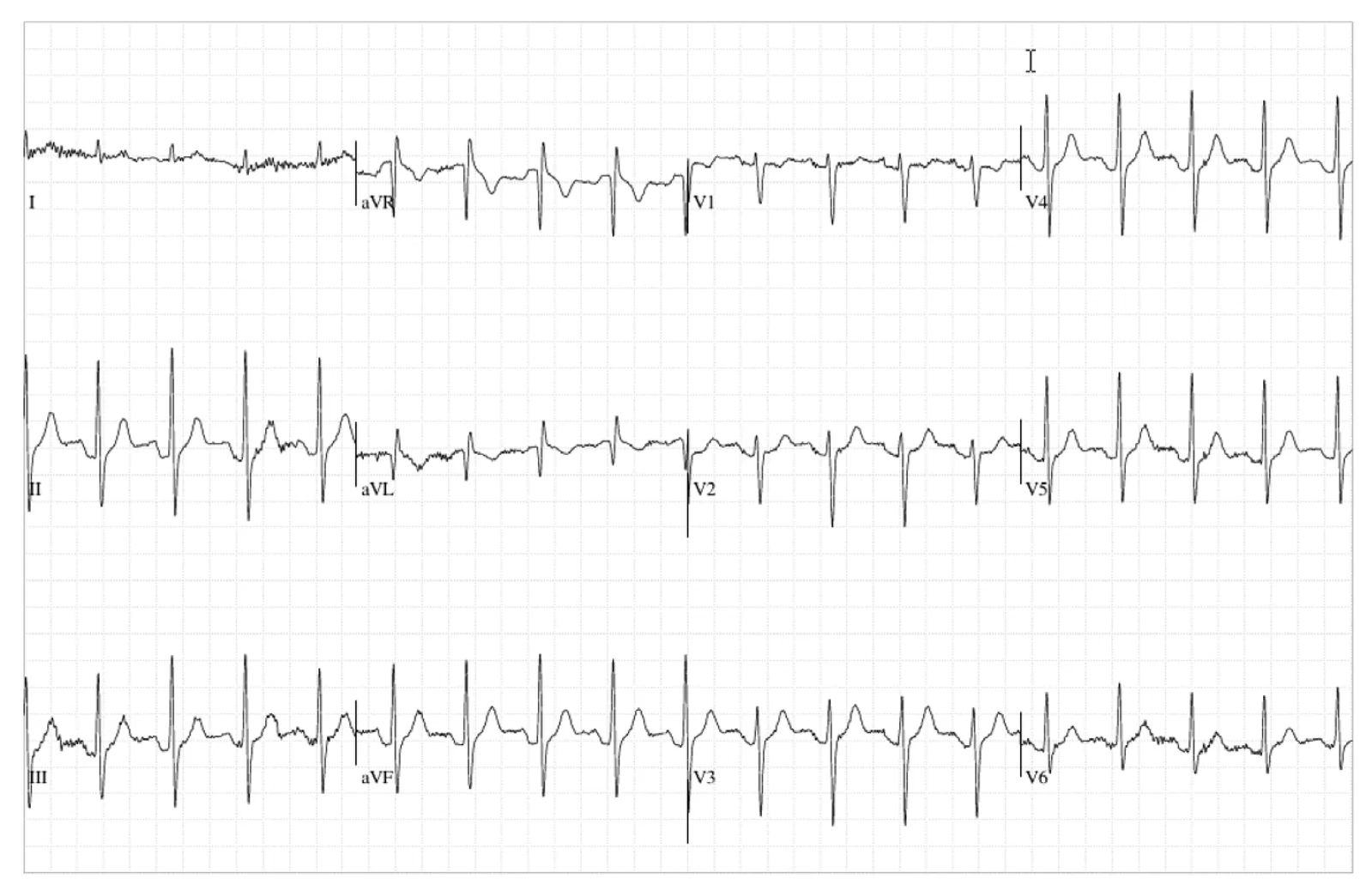
Twelve-lead ECGs obtained during an exercise test performed 4 months after the first episode of CHB and off propranolol (March 2023). The first ECG, recorded at baseline, shows sinus rhythm at 80–85 bpm, with a QT interval of 280 ms and a corrected QT (QTc) of 385 ms. The second ECG, obtained at peak exercise, shows a heart rate of 175–180 bpm with 1:1 AV conduction, a QT interval of 240–260 ms, and a QTc of 400 ms. The final ECG, recorded during recovery (3–4 min after peak exercise), shows sinus rhythm at a heart rate of 111 bpm, with a QT interval of 260 ms and a QTc of 360 ms. The corrected QT intervals were calculated using Bazett’s formula.

**Figure 7:**
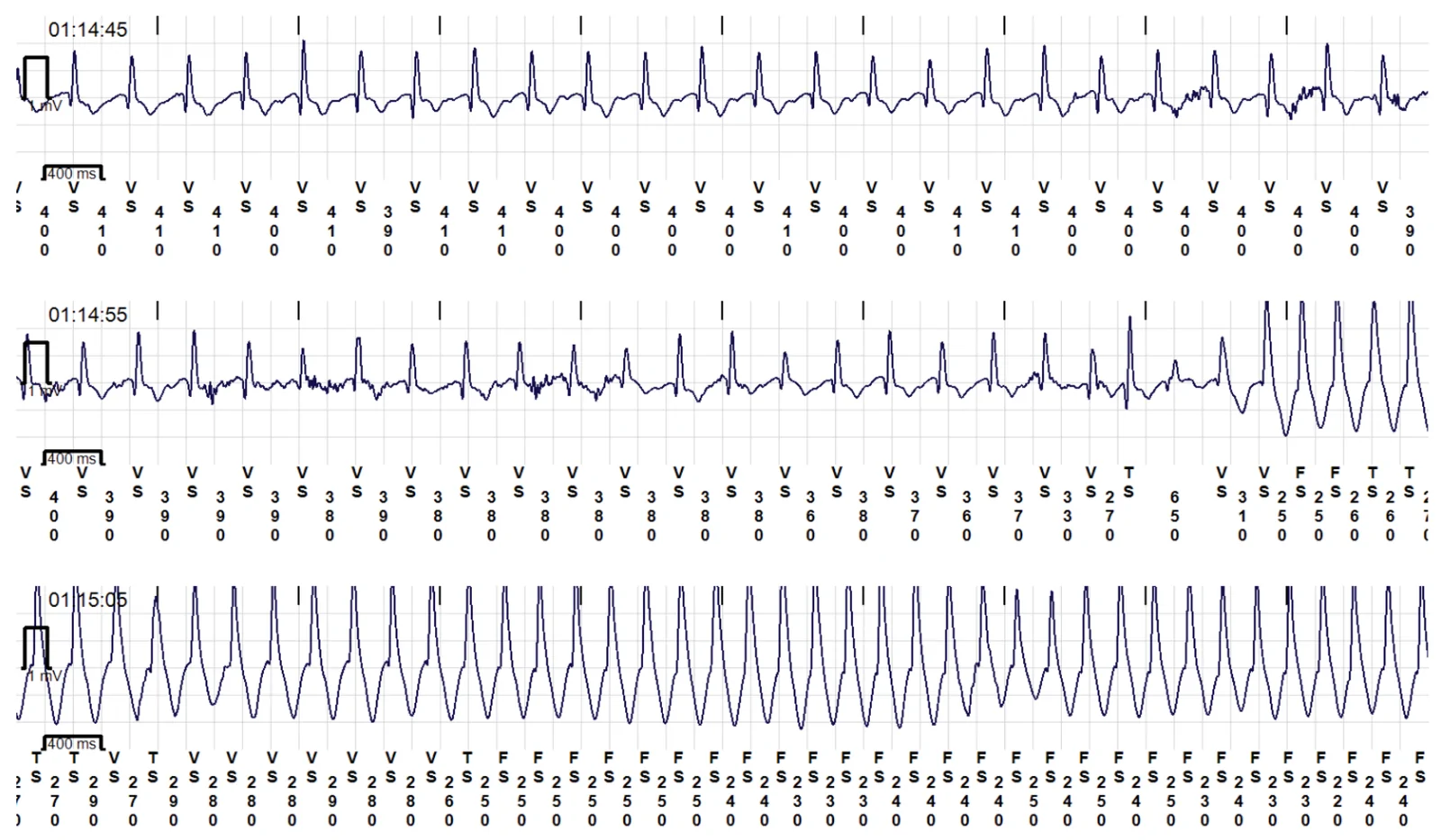
Implantable loop recorder (ILR) tracing during an episode of wide-complex tachycardia with a rate of 240–260 bpm. Abrupt onset, with possible initial fusion beats, increases the likelihood of monomorphic ventricular tachycardia. However, interpretation is limited by the single-channel ILR recording and the inability to definitively assess AV dissociation. (September 2024).

**Figure 8:**
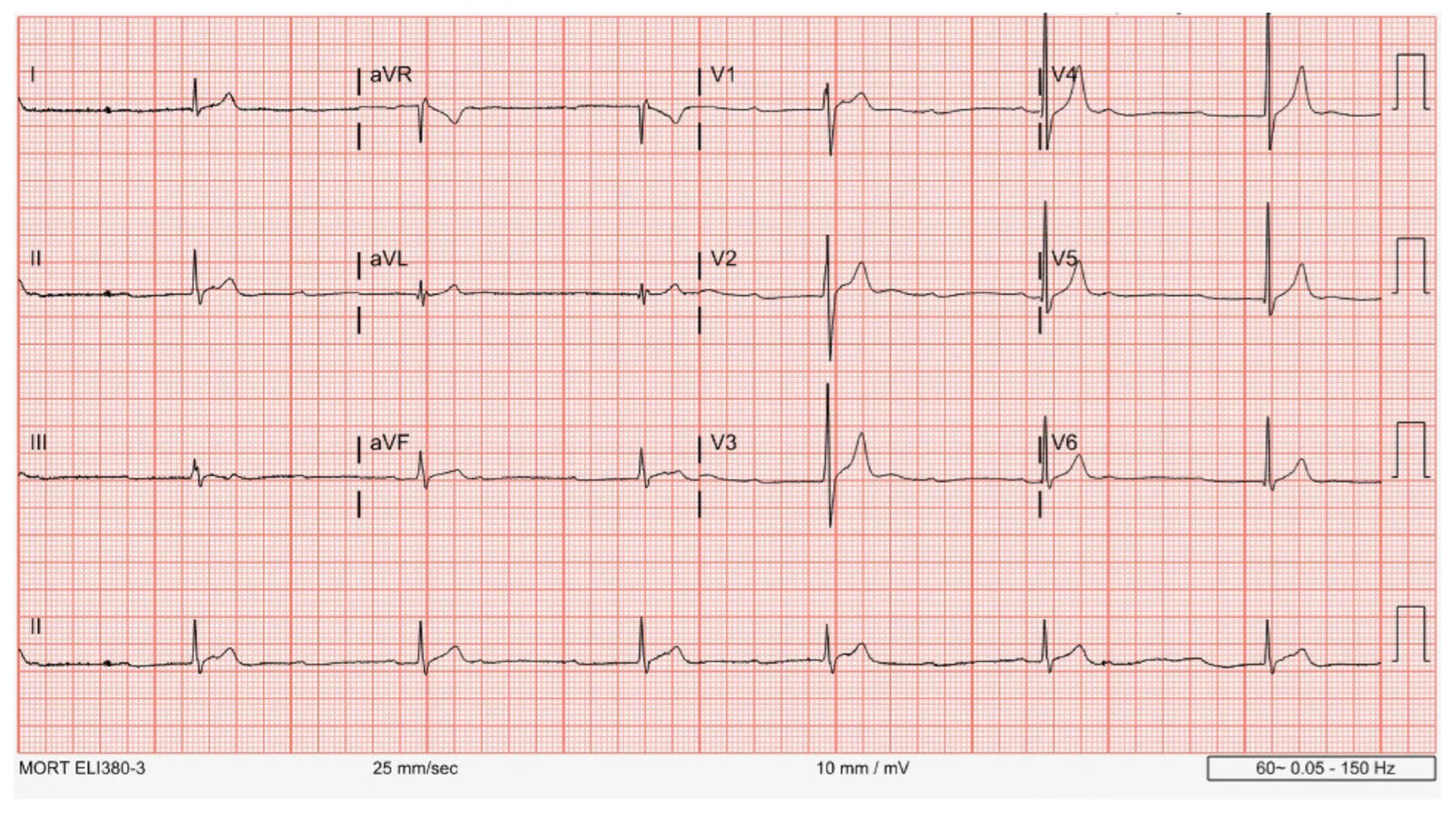
A 12-lead ECG with CHB with a slow junctional escape rhythm at 40 bpm (September 2024).

A second exercise test was performed in March 2024 while the patient remained off propranolol, again demonstrating normal AV conduction and an appropriate QT response at maximal heart rate and during recovery (Figure 6). Propranolol was reintroduced in April 2024 at a reduced dose; however, the patient developed symptomatic bradycardia, resulting in poor tolerance and adherence. In early September 2024, the ILR recorded a 3-min episode of sustained wide-complex tachycardia during exercise, interpreted as either ventricular tachycardia or supraventricular tachycardia with aberrant conduction, prompting hospitalization (Figure 7). The patient experienced palpitations and presyncope but did not lose consciousness. Definitive classification was not possible because the event was recorded using a single-channel ILR. A few days later, the patient developed recurrent paroxysmal and symptomatic CHB with a junctional escape rhythm of approximately 40 bpm (Figure 8).

Given the recurrence of conduction disease, the need for ongoing antiarrhythmic therapy, the actual genotype, and the occurrence of possible ventricular arrhythmia, a decision was made to implant a transvenous dual-chamber ICD. The device was programmed in AAIR–DDDR mode, with a lower rate of 60 bpm and an upper tracking rate of 150 bpm. Propranolol was subsequently replaced with nadolol due to better tolerability.

At the most recent follow-up in August 2025, the patient weighed 108.3 kg and measured 195.4 cm in height. He was clinically stable on nadolol, with intermittent atrial tachycardia recorded on device interrogation, predominantly preserved AV conduction, and a low percentage of ventricular pacing. Transthoracic echocardiography demonstrated normal left ventricular systolic function.

Familial segregation analysis revealed that the patient’s father carries the *SCN5A* variant. He has a history of atrial fibrillation before 40 years and previously mild left ventricular systolic dysfunction on cMRI, which subsequently normalized on follow-up on echocardiography without heart failure therapy. His ECG shows normal AV conduction, no Brugada pattern, and isolated monomorphic premature ventricular complexes (PVCs). Exercise stress testing was normal, and 24-h Holter monitoring demonstrated monomorphic PVCs accounting for <1% of total beats. He was not any medication. The patient’s mother and sister carry the *KCNH2* variant and are phenotype-negative, with normal ECGs and no QT prolongation. There are no other first-degree siblings. Extended family screening is ongoing, and there is no known family history of sudden cardiac death or early-onset cardiac disease.

## 3. Discussion

We present a patient with an atypical and complex arrhythmic phenotype, characterized by early-onset of atrial flutter and tachycardia, followed by intermittent CHB, sinus node dysfunction, and an episode of sustained monomorphic wide-complex tachycardia. This was accompanied by transient mild left ventricle dysfunction. Genetic testing identified two heterozygous pathogenic variants: *SCN5A* (NM_003335.5: c.656G>A; p.Arg219His), previously implicated in arrhythmia and dilated cardiomyopathy, and *KCNH2* (NM_000238.4: c.2717C>T; p.Ser906Leu), associated with LQT2. Given the recurrence of conduction disease, the need for ongoing antiarrhythmic therapy, and the occurrence of possible ventricular arrhythmia, a transvenous dual-chamber ICD with pacing capability was implanted.

The *SCN5A* p.Arg219His variant is rare (allele frequency ~0.0002% in exomes, ~0.0007% in genomes and ~0.0002% in total) in gnomAD v4.1.1 and meets the criteria for pathogenic according to ACMG guidelines based on its low allele frequency (PM2), functional evidence, and supportive in silico predictions. [4]. Functional studies demonstrated that this variant induces a gating pore proton leak, representing a mechanism that disrupts electrical stability and myocardial contractile performance (PS3) [5,6,7]. In silico prediction tools consistently support a deleterious effect (PP3) [8]. In ClinVar, there is one submission classifying the variant as of uncertain significance (VUS) and seven as likely pathogenic or pathogenic, with reported associations including atrial and ventricular arrhythmias, sinus node dysfunction, atrial standstill, progressive conduction disease, Brugada syndrome, and dilated cardiomyopathy [9]. Prior reports describe marked phenotypic variability and incomplete penetrance, with manifestations spanning conduction system disease, atrial and ventricular arrhythmias, and cardiomyopathy [5,6,10].

The *KCNH2* p.Ser906Leu variant is slightly more frequent, but still rare (allele frequency ~0.008% in exomes, ~0.004% in genomes and ~0.008% in total) in gnomAD v4.1.1 and meets the criteria for likely pathogenic according to ACMG guidelines based on its low allele frequency (PM2), functional evidence, and supportive in silico predictions. [11]. Functional studies have demonstrated a reduction in rapid delayed rectifier potassium current (I_Kr_) with moderate hERG channel loss-of-function, consistent with the molecular mechanism underlying LQT2 (PP2) [12]. In silico predictions are mixed (PP3), and ClinVar submissions show variable classification with seven descriptions as VUS and seven descriptions as pathogenic or likely pathogenic [13,14]. Importantly, clinical data suggest low penetrance. A study of eight Dutch families carrying this variant reported highly variable expressivity and incomplete penetrance, supporting its classification as a low-penetrance pathogenic LQT2 variant [12].

Digenic or compound heterozygous variants in Long QT syndrome (LQTS) associated genes are identified in approximately 4–11% of cases and are typically associated with more severe phenotypes, including early-onset QT prolongation, sinoatrial node dysfunction, and malignant ventricular arrhythmias [15,16,17]. Previously described cases involving digenic *SCN5A*–*KCNH2* variants have largely presented with severe LQTS phenotypes and early sinus node dysfunction [17,18,19,20]. In contrast, our patient exhibited a distinct clinical phenotype, lacking features of severe LQTS, isolated LQT2 or classic Brugada syndrome. He exhibited neither spontaneous nor drug-induced Brugada ECG patterns and the QT interval remained normal throughout follow-up, including during periods of sinus rhythm and during exercise testing.

The observed phenotype, dominated by atrial arrhythmias, progressive conduction disease, low-voltage P waves (Figure 2), and intermittent high-grade AV block, falls well within the recognized spectrum of SCN5A-related disease, which is known for marked phenotypic variability. Therefore, the SCN5A p.Arg219His variant is likely a major contributor or even the only contributor to the phenotype.

As shown in functional studies by Gosselin-Badaroudine et al. 2012 [5], the SCN5A p.Arg219His variant creates an abnormal gating pore within the voltage-sensing domain of Nav1.5, allowing an aberrant proton leak current independent of the central sodium-conducting pore. This represents a gain of abnormal function rather than a conventional alteration of peak sodium current. Intracellular acidification caused by this proton leak is thought to impair intracellular calcium homeostasis and excitation–contraction coupling, providing a mechanistic explanation for the dilated cardiomyopathy phenotype associated with this variant. In addition, intracellular acidification may impair gap junction coupling and slow electrical conduction, thereby contributing to the conduction disease and atrial arrhythmias observed in affected families. Thus, although the underlying biophysical defect represents a gain of abnormal function, the resulting clinical phenotype shares many features with SCN5A loss-of-function disorders [1,5,21].

The intermittent nature of the patient’s complete heart block, characterized by preserved AV conduction between episodes, including during exercise testing at high heart rates, together with normal AH and HV intervals during both electrophysiological studies, argues against fixed conduction system disease. Similarly, the absence of late gadolinium enhancement or imaging features suggestive of arrhythmogenic cardiomyopathy on cardiac magnetic resonance imaging argues against an underlying structural myocardial substrate. Together, these findings support a dynamic rather than fixed conduction system dysfunction, likely multifactorial in origin. During these episodes, other reversible causes of AV block, including myocarditis, infectious etiologies and hypothyroidism, were investigated and rapidly excluded based on laboratory testing and the clinical presentation. Although beta-blocker therapy may have contributed to or exacerbated the conduction abnormalities, no consistent temporal relationship was observed between beta-blocker administration and episodes of high-grade AV block. Furthermore, first-degree AV block was documented before initiation of propranolol therapy, arguing against a purely pharmacological mechanism. The absence of AV block during the initial electrophysiological study and ablation, which preceded the first episode of complete heart block, together with stable AH and HV intervals throughout the procedure and recurrence of AV block one year later, further argues against a procedure-related etiology.

The mild left ventricular systolic dysfunction observed in both the proband and his father, who carry the SCN5A p.Arg219His variant, may be attributable to this variant and could represent an early or mild manifestation of the associated cardiomyopathic phenotype. Although ventricular function subsequently normalized in both individuals, these findings could be consistent with previous reports linking p.Arg219His to an early form of SCN5A-associated dilated cardiomyopathy through the gating pore proton leak mechanism [1,21]. Another possible explanation for the proband’s mild left ventricular dysfunction is tachycardia-induced cardiomyopathy related to the initial high burden of atrial arrhythmias before treatment initiation.

While the conduction abnormalities, atrial arrhythmias, mild ventricular dysfunction, and possible ventricular arrhythmia are most consistent with the SCN5A variant, a modifying effect of the KCNH2 variant on disease expression cannot be excluded. Nevertheless, no overt LQT2 phenotype was not observed in this patient. Although prolonged periods of atrial flutter and atrial tachycardia are associated with rate-dependent shortening of ventricular repolarization and may reduce the likelihood of detecting QT prolongation, QT intervals remained within normal limits after restoration of sinus rhythm and during both exercise testing. Taken together, these findings suggest that this low-penetrance, variably expressive KCNH2 variant was not the primary driver of the observed phenotype.

Significant phenotypic differences were observed between the father and the proband, with the latter developing a more severe clinical presentation despite sharing the same *SCN5A* variant. This variability may reflect incomplete penetrance of the variant itself but could also be influenced by additional genetic, environmental (e.g., beta-blocker therapy or competitive athletic activity), epigenetic, or age-related factors that modify disease expression. The possibility that the *KCNH2* p.Ser906Leu variant also contributed to the phenotypic differences between the father and the proband cannot be excluded.

The absence of characteristic electrocardiographic features of either Brugada syndrome or LQT2, despite the presence of pathogenic variants in both SCN5A and KCNH2, further highlights the challenges of genotype–phenotype correlations in inherited channelopathies and the limitations of predicting clinical severity based solely on genotype.

The decision to implant a transvenous dual-chamber ICD with pacing capability was individualized and reached through multidisciplinary team consensus after consultation with experts from other centers, given the absence of clear guideline recommendations for this specific clinical scenario. This consensus was based on the patient’s recurrent symptomatic conduction disease, possible ventricular arrhythmia, and uncertain overall arrhythmic risk profile. Although the mechanism of the wide-complex tachycardia could not be established with certainty, ventricular tachycardia could not be excluded. Considering these factors, together with the potential for ventricular arrhythmias associated with the SCN5A p.Arg219His variant and the uncertain contribution of the coexisting KCNH2 variant, a dual-chamber ICD was favored over a pacemaker alone.

## 4. Conclusions

This case illustrates a rare and complex arrhythmic phenotype in a pediatric patient carrying digenic heterozygous arrhythmogenic variants in *SCN5A* p.Arg219His and *KCNH2* p.Ser906Leu. The atypical phenotype was most likely driven predominantly by the *SCN5A* p.Arg219His variant, while the contribution of the low-penetrance *KCNH2* variant to the patient’s phenotypic expression remains uncertain.

This case highlights the challenges of genotype–phenotype correlations in inherited arrhythmia syndromes and underscores the need for cautious, phenotype-driven clinical decision-making, even in the presence of established disease-causing variants. Although variants of uncertain significance and low-penetrance pathogenic variants may be particularly difficult to interpret, this case illustrates that even well-established pathogenic variants can pose significant diagnostic and therapeutic challenges when associated with an atypical phenotype. Ultimately, while genetic findings are essential for risk assessment and clinical management, therapeutic decisions should be guided primarily by the patient’s phenotype, with genotype providing complementary information, particularly when considering invasive procedures or device implantation. Additional reports of similar digenic cases will be required to better define the clinical significance of this genetic combination.

## Data Availability

The data presented in this study are available from the corresponding author upon reasonable request. The data are contained within the patient’s hospital medical record and can be made available to reviewers if requested. They are not publicly available due to patient privacy considerations and applicable provincial and federal privacy regulations in Quebec, Canada.

## Supplementary Materials

The following supporting information can be downloaded at: https://www.mdpi.com/article/10.3390/genes17080885/s1.

## Abbreviations

The following abbreviations are used in this manuscript:

AV	Atrioventricular
AH	Atrial-His
CHB	Complete heart block
CNV	Copy number variants
EPS	Electrophysiology study
HV	His-ventricular
ICD	Implantable cardioverter-defibrillator
InDels	Insertion/deletions
ILR	Implantable loop recorder
LQTS	Long QT syndrome
LQT2	Long QT syndrome type 2
LQT3	Long QT syndrome type 3
cMRI	Cardiac magnetic resonance imaging
NGS	Next-generation sequencing
SNV	Single nucleotide variants
TEE	Transesophageal echocardiography
