# Supplementary material for: A Complex Arrhythmic Phenotype in a Pediatric Patient with Variants in SCN5A and KCNH2
Source: Genes (Basel). 2026 Jul 29;17(8):885. doi: 10.3390/genes17080885 (PMC13512050; doi:10.3390/genes17080885)
Supplement: Supplementary file 1 [file genes-17-00885-s001.zip › genes-4391611-supplementary.pdf]

Supplementary document

In 2024 the molecular analysis of the *unexplained cardiac arrest* panel performed for our patient by the molecular diagnostic laboratory of the *Institut de cardiologie de Montréal* includes the detection of single nucleotide variants (SNV), small insertions/deletions (InDel), and copy number variants (CNV) in the following 73 genes:

AARS2(NM_020745.3), ACTC1(NM_005159.4), ACTN2(NM_001103.3), ALPK3(NM_020778.5), ANK2(NM_001148.5), BAG3(NM_004281.3), CACNA1C(NM_199460.3), CALM1(NM_006888.4), CALM2(NM_001743.5), CALM3(NM_005184.4), CASQ2(NM_001232.3), CDH2(NM_001792.5), CSRP3(NM_003476.4), DES(NM_001927.3), DMD(NM_004006.2;rs587776747,rs398124091,rs797045526,rs398124099), DOLK(NM_014908.3), DSC2(NM_024422.4), DSG2(NM_001943.4), DSP(NM_004415.3), EMD(NM_000117.2), FHL1(NM_001159702.2), FHOD3(NM_001281740.3), FKRP(NM_024301.5), FKTN(NM_001079802.1;rs1554754182), FLNC(NM_001458.4), GLA(NM_000169.2;rs199473684), GNB5(NM_016194.4),HCN4(NM_005477.2), JPH2(NM_020433.4), JUP(NM_002230.3), KCNE1(NM_001127670.3), KCNE2(NM_172201.1), KCNH2(NM_000238.3), KCNJ2(NM_000891.2), KCNQ1(NM_000218.2), LAMP2(NM_001122606.1), LMNA(NM_170707.3), MYBPC3 (NM_000256.3;rs36212066,rs886041030,rs397515893,rs1114167419,rs587776699,rs786204336,rs1025692267,rs864622197 ,c.1090+453C>T,c.2731_2737+19del,c.1091-575A>C,c.1224-21A>G,), MYH7(NM_000257.3), MYL2(NM_000432.3), MYL3(NM_000258.2), NEXN(NM_144573.3), NKX2-5(NM_004387.4), NRAP(NM_198060.3), PKP2(NM_001005242.2), PLN(NM_002667.4), PPA2(NM_176869.3), PPCS(NM_024664.4), PPP1R13L(NM_006663.4), PRKAG2(NM_016203.3), PTPN11(NM_002834.4), RAF1(NM_002880.3), RBM20(NM_001134363.2), RIT1(NM_006912.6), RYR2(NM_001035.2), SCN5A(NM_198056.2), SGO1(NM_001199251.3), SLC4A3(NM_005070.3), TAFAZZIN(NM_000116.4), TBX5(NM_181486.4), TECRL(NM_001010874.4), TMEM43(NM_024334.2), TNNC1(NM_003280.2), TNNI3(NM_000363.4), TNNI3K(NM_015978.2), TNNT2(NM_001001430.2), TPM1(NM_001018005.1), TRDN(NM_006073.3), TRIM63(NM_032588.4), TRPM4(NM_017636.3), TTN(NM_001267550.2), TTR(NM_000371.3), VCL(NM_014000.2)
